# Emotional, Social and Occupational Adjustment among Oncology Nurses

**Published:** 2016-10-01

**Authors:** Mohammad Vaezi, Mahboubeh Vala, Maryam Souri, Ashraf Mousavi, Ardeshir Ghavamzadeh

**Affiliations:** Hematology-Oncology and Stem Cell Transplantation Research Center, Tehran University of Medical Sciences, Tehran, Iran

**Keywords:** Oncology nurses, Social, Occupational, Emotional, Adjustment

## Abstract

**Background** **:** Social, occupational and emotional adjustment of Oncology nurses were assessed and compared with other nurses in this study.

**Subjects and Methods:** One hundred nurses including Oncology nurses (n=50) and non-Oncology nurses (n=50) participated in cross-sectional study conducted in Shariati Hospital. Bell's Adjustment Inventory was used to measure social, emotional and occupational adjustment. Survey data were entered into SPSS statistical software, version 18 and the Kruskal-Wallis test was used for data analysis.

**Results:** The study included nurses from Women’s Internal Medicine ward (14%), Men’s Internal Medicine ward (13%); Midwifery unit (17%), Operating room (15%) and Hematology-Oncology ward (41%). The mean age of the participants was 36.98 ± 8.28 years. In group of Hematology-Oncology nurses, the mean scores for occupational, social and emotional adjustment were 13.23 ± 1.99, 12.47 ± 1.79 and 18.19 ± 2.52, respectively. Data analysis showed that there is a statistically significant difference in the mean score of three areas of adjustment between Oncology nurses and their colleagues working in general wards (p-value=0.002, p-value<0.001, p-value<0.001 for occupational, social and emotional adjustment, respectively).

**Conclusion:** The results of the study indicated that Oncology nurses had significantly lower social, occupational and emotional adjustment compared with nurses working in other wards.

## Introduction

 Human resources are considered as the most important asset of an organization and the quality of human resources may have a great influence on success, survival and promotion of organization. Nowadays, work-related tensions are prevalent among people, typically affecting adjustment of those people working as health and education experts and other people who work in public welfare responsibilities such as nurses.^1^

According to Dawis and Lofquist, people enter their needs into the work environment and in return working environment has demands from employees. Two key elements in this theory are work adjustment and environmental structure. Work adjustment is ideal when person and environment have matching work needs and work skills. A worker's attempts to improve his/her fitness to work environment can be considered as actions designed to achieve work adjustment.^2^

The basic assumption in the theory of work adjustment is that each individual employee seeks to achieve and maintain a positive relationship with their work environment.^3^ Work adjustment is an important factor in individual’s successful continuation in work setting. Various definitions of work adjustment have been offered, one of which is defined as compatibility between an individual and a work environment.^4^ Emotional adjustment may include good mental health, personal life satisfaction and coordination between feelings, activities and thoughts, i.e. emotional adjustment is the mechanism by which individual achieves emotional stablility.^5^

Social adjustment is a process by which the relationships between persons, groups and cultural elements are established on a mutually satisfactory basis.^6^ In fact, work-related tensions and poor fit to the work environment may result in expensive costs to employees and organizations including less efficiency, increased job withdrawals, development of physical and mental disorders, repeated sick leave requests and increase in number of events during work, weak performance of personnel and reduction in job satisfaction^7^^,^^8^ Among the 130 occupations evaluated by the Institute for Occupational Health and Safety, nursing was ranked as 27 with regard to mental health problems.^9^

Nursing is very stressful job.^10^ Nurses regularly come face to face with patients suffering from incurable diseases and continuously experience severe stress.^11^ Working conditions and environments have considerable effect on nurses’ attitude toward work environment as well as emotional and social adjustment.^12^

Working in an Oncology ward and providing nursing care to cancer patients is stressful for nurses.^13^ Oncology nurses are exposed to extraordinary pressures, abnormal amount of suffering and death, treatment-related side effects and long-term patient care compared to their colleagues working in different care settings.^14^ Over 70% of Oncology nurses experience mild to moderate emotional exhaustion and job burnout.^15^ Job burnout associated with long-term work stress can leave nurses vulnerable to emotional problems in comparison with their colleagues working in outpatient settings.^16^ In Hooper et al. study, other factors such as patient mortality were the most common causes of emotional exhaustion in nurses and physicians.^17^ Similarly, it has been reported that there is a significant relationship between emotional factors such as mortality of patients and emotional exhaustion. Evidence indicates that more exposure to patients' deaths has been linked to higher reports of stress and burnout in nurses.^18^ conversely, when nurses are satisfied, positive patient outcomes have been known to increase. In order to give a safe and effective care, the nurses, especially Oncology nurses, should keep an appropriate balance between the personal and professional aspects of their life. The importance of this study is that nurses need to have mental health to provide the highest quality of care to patients. The purpose of this study was to compare the emotional, social and job adjustment between nurses working in Oncology wards and their colleagues working in different care settings.

## SUBJECTS AND METHODS

 One hundred nurses working in Women's Internal Medicine ward, Men's Internal Medicine ward, Operating room and Medical Oncology ward were enrolled in this cross-sectional study conducted in Shariati Hospital in 2013. This study was approved by the Ethics Committee of our institute. All participants were strictly volunteers. Criteria for inclusion into this study were as follows:

1) being a registered nurse 2) female gender and 3) having at least one year of experience as head nurse, staff nurse and licensed practical nurse.

**Data collection tool**

Those volunteers who met the study criteria (n=100) were asked to complete Bell's Adjustment Inventory (BAI) questionnaire. Throughout this study, all responses to survey questions remained  confidential. The participants were made aware that they were free to withdraw at any time. Bell's Social, Emotional and Occupational Adjustment Inventory questionnaire was used for collection of data. This questionnaire was adapted from Bell's Adjustment Inventory developed in 1961.

Two forms of Bell’s Adjustment Inventory were available: one for students and one for adults. The inventory was used to measure adjustments in the areas of home, health, emotional, occupational and social. The coefficient of reliability for each component was 0.91, 0.81, 0.91, 0.85 and 0.88, respectively. Three of 5 areas emotional, occupational and social were explored in this study.

**Occupational adjustment**

A high score indicates high job  satisfaction and low score indicates low job satisfaction.

**Emotional adjustment**

Study participants who score high are emotionally unstable individuals but those scoring low are described emotionally stable.

**Social adjustment**

Participants who achieve high scores are socially isolated and participants with low scores show aggression in social interactions.^19^ In Iran, a number of researchers have reported the reliability and validity of this questionnaire.^20^

**Statistical analysis**

The Morgan analysis was used to determine the appropriate sample size for survey. According to Table 1, N stands for population size (N=117) and S stands for sample size (S=100). The collected data were analyzed using SPSS 18, descriptive statistics, T-tests and the Kruskal-Wallis tests.

## Results

 The study included nurses from Women’s Internal Medicine ward (14%), Men’s Internal Medicine ward (13%), Midwifery unit (17%), Operating room (15%) and Oncology ward (41%). The mean age of the participants was 36.98 ± 8.28 years. The majority of nurses in Women's Internal Medicine ward were less than 30 years of age (50%), while in other wards including Men's Internal Medicine ward, Midwifery unit, Operating room and Oncology ward were 30-40 years old (50%, 44%, 50% and 40%, respectively). In women's Internal Medicine ward, most of nurses had maximum of 1-5 years of experience, while it was 10-15 years in Men's Internal Medicine ward, 1-5 years in Midwifery unit, 5-15 years in Operating room and 5-15 years in Oncology wards. Table 1 demonstrates sex distribution of participants.

Overall mean score for occupational, social and emotional adjustment was 14.22 ± 2.26, 13.64 ± 2.11 and 20.94 ± 2.92, respectively. In the group of Oncology nurses, the scores were 13.23 ± 1.99, 12.47 ± 1.79 and 18.19 ± 2.52, respectively.

**Table 1:** Sex distribution of nurses in different wards

**Nursing wards**	**Male (%)**	**Female (%)**	**Total (%)**
**Internal Medicine ward (Women)**	-	14 (100)	14 (100)
**Internal Medicine ward (Men)**	13 (100)	-	13 (100)
**Midwifery unit**	-	17 (100)	17 (100)
**Operating room**	8 (100)	7 (100)	15 (100)
**Oncology**	3 (100)	38 (100)	41 (100)

Data analysis showed that there is a statistically significant difference in the mean score of three areas of adjustment between nurses working in Oncology wards and their colleagues working in general wards (Table 2). Also, nurses assigned to Internal Medicine Women's ward showed the highest average scores of emotional, social and occupational adjustment compared to Oncology nurses. There is a statistically significant difference in three areas of adjustment between nurses working in Oncology wards and those working in other different care settings.

## Discussion

 In this study, we compared the emotional, occupational and social adjustment between Oncology and non-Oncology nurses. The study revealed that working conditions and environment play a critical role in mental health of nursing staff and their adjustment. This study is unique and results of our inquiry can be applied to improve the mental health of nursing staff and quality of cancer care. The results obtained in this survey were found to be similar to those of previous studies.

According to these findings, Oncology nurses experience higher levels of stress due to more morbidity and death of patients, more patients’ treatment-related side effects, long-term patient care and more exposure to patients with pain and suffering compared to their colleagues working in different care settings. Over 70% of Oncology nurses experience mild to moderate emotional exhaustion and job burnout.^13^^-^^16^

**Table 2:** Average rating, Kruskal-Wallis test

**Adjustment variables**	**Group**	**N (%)**	**Standard deviation**	**mean**	**p-value**
**Emotional adjustment**	Internal Medicine ward (Women)	14	± 1.91	26.50	0.002
	Internal Medicine ward (Men)	13	± 2.92	21.40	
	Midwifery unit	17	± 2.86	23.05	
	Operating room	15	± 1.82	24.80	
	Oncology and Bone Marrow Transplantation	41	± 2.52	18.19	
**Social adjustment**	Internal Medicine ward (Women)	14	± 1.64	16.28	<0.001
	Internal Medicine ward (Men)	13	± 1.24	13.33	
	Midwifery unit	17	± 2.11	15.80	
	Operating room	15	± 2.16	14.76	
	Oncology and Bone Marrow Transplantation	41	± 1.79	12.47	
**Occupational** **adjustment**	Internal Medicine ward (Women)	14	± 1.77	17.28	<0.001
	Internal Medicine ward (Men)	13	± 2.04	14.82	
	Midwifery unit	17	± 1.93	16.00	
	Operating room	15	± 2.33	14.76	
	Oncology and Bone Marrow Transplantation	41	± 1.99	13.23	

*This Table shows statistically significant differences in mean scores of three areas of adjustment between nurses working in Oncology and non-Oncology settings

In another study, other factors such as patient mortality were the most common causes of emotional exhaustion in nurses and physicians.^17^

The result of their study is consistent with our study which demonstrates Oncology nurses experience less emotional adjustment. The results of our study indicated that Oncology nurses had significantly lower social, occupational and emotional adjustment compared to other nurses.

## CONCLUSION

 The study has indicated that Oncology nurses experience more difficulties, resulting in negative effects on their emotional, social and occupational adjustment; therefore, special attention must be paid to improve their mental health status. We hope this study will, by understanding of stressful working environments of nurses, reduce their vulnerabilities.

## References

[1] Kuusio H, Heponiemi T, Sinervo T (2010). Organizational commitment among general practitioners: a cross-sectional study of the role of psychosocial factors. Scand J Prim Health Care.

[2] Huang FF, Yang HH (2011). The effects of nationality differences and work stressors on work adjustment for foreign nurse aides. BMC Health Serv Res.

[3] Iacovides A, Fountoulakis KN, Moysidou C (1999). Burnout in nursing staff: is there a relationship between depression and burnout?. Int J Psychiatry Med.

[4] Kuerer HM, Eberlein TJ, Pollock RE (2007). Career satisfaction, practice patterns and burn out among surgical oncologists: Report on the quality of life of members of the society of surgical oncology. Ann Surg Oncol.

[5] Sherman DW, Haber J, Hoskins CN (2009). Differences in physical, emotional, and social adjustment of intimate, family, and nonfamily patient-partner dyads based on a breast cancer intervention study. Oncol Nurs Forum.

[6] Weissman MM, Sholomskas D, John K (1981). The assessment of social adjustment. An update. Arch Gen Psychiatry.

[7] Utriainen K, Kyngäs H (2009). Hospital nurses' job satisfaction: a literature review. J Nurs Manag.

[8] Coomber B, Barriball KL (2007). Impact of job satisfaction components on intent to leave and turnover for hospital-based nurses: a review of the research literature. Int J Nurs Stud.

[9] Rastegari M, Khani A, Ghalriz P (2010). Evaluation of quality of working life and its association with job performance of the nurses. Iran J Nurs Midwifery Res.

[10] Najimi A, Moazemi Goudarzi A, Sharifirad G (2012). Causes of job stress in nurses: A cross-sectional study. Iran J Nurs Midwifery Res.

[11] Adams A, Bond S (2000). Hospital nurses' job satisfaction, individual and organizational characteristics. J Adv Nurs.

[12] Wennman-Larsen A, Persson C, Ostlund U (2008). Development in quality of relationship between the significant other and the lung cancer patients perceived by the significant other. Eur J Oncol Nurs.

[13] De Carvalho EC, Muller M, de Carvalho PB (2005). Stress in the professional practice of oncology nurses. Cancer Nurs.

[14] Delvaux N, Razavi D, Marchal S (2004). Effects of 105 hours psychological training program on attitudes, communication skills and occupational stress in oncology: a randomised study. Br J Cancer.

[15] Amiresmaili M, Moosazadeh M (2013). Determining job satisfaction of nurses working in hospitals of Iran: A systematic review and meta-analysis. Iran J Nurs Midwifery Res.

[16] Barrett L, Yates P (2002). Oncology/haematology nurses: a study of job satisfaction, burnout, and intention to leave the specialty. Aust Health Rev.

[17] Hooper C, Craig J, Janvrin DR (2010). Compassion satisfaction, burnout, and compassion fatigue among emergency nurses compared with nurses in other selected inpatient specialties. J Emerg Nurs.

[18] Peters L, Cant R, Payne S (2013). How Death Anxiety Impacts Nurses’ Caring for Patients at the End of Life: A Review of Literature. Open Nurs J.

[19] Barnes TC, Amoroso MD (1947). Electroencephalograms correlated with scores of the Bell adjustment inventory for personality. Fed Proc.

[20] Dehestani M, Tarkhan M, Abbasi M (2012). Efficacy of integrating stress coping skills training with detoxification on social adjustment of addicted women. Addict Health.

